# Mental health education in undergraduate medical curricula across Nepalese universities

**DOI:** 10.1186/s12909-021-02743-3

**Published:** 2021-05-28

**Authors:** Kedar Marahatta, Sagun Ballav Pant, Madhur Basnet, Pawan Sharma, Ajay Risal, Saroj Prasad Ojha

**Affiliations:** 1 WHO Country Office for Nepal, Kathmandu, Nepal; 2 Department of Psychiatry and Mental Health, Institute of Medicine, Tribhuvan University, Kathmandu, Nepal; 3 Department of Psychiatry BP, Koirala Institute of Health Sciences, Dharan, Nepal; 4 Department of Psychiatry, Patan Academy of Health Sciences, Lalitpur, Nepal; 5 Department of Psychiatry, Dhulikhel Hospital, Kathmandu University School of Medical Sciences, Dhulikhel, Kavre, Nepal

**Keywords:** Mental Health, Curriculum, Medical Education, Medical Students, Task sharing, Nepal

## Abstract

**Background:**

Mental and substance use disorders account for 30 % of the non-fatal disease burden and 10 % of the overall disease burden but the treatment gap is daunting. With just one psychiatrist per 200,000 populations in Nepal, the only convincing way to improve access to the services quickly is by mobilizing non-specialized medical practitioner. A robust mental health component within the training curriculum of general medical doctors could produce medical graduates with adequate knowledge and skills to deliver basic mental health service. We reviewed the mental health curricula for medical students of all the medical universities in Nepal.

**Methods:**

Information on existing mental health curricula was collected from the faculty of the respective universities with respect to content coverage, teaching methods and evaluation patterns. The mental health curricula were described in relation to teaching duration, duration of clinical rotation, duration of internship, and the relative weight of mental health in examination marks. Teaching methods were classified broadly as passive and active. Assessment methods were documented. Content coverage of the curricula was evaluated with respect to history taking and general physical examination, the priority mental health conditions, topics on behavioural sciences, and child mental health or other topics.

**Results:**

The duration of teaching on mental health in general medical doctor training in Nepalese medical universities ranges from 25 to 92 h. All medical universities have a relative focus on the priority mental neurological and substance use disorders. The clinical rotation on mental health is mostly two weeks, except in one university where it can be extended up to 4 weeks with an elective clinical rotation. The relative weight of summative assessment ranges from 0.21 to 2.5 % total marks of the entire training.

**Conclusions:**

Considerable disparities exist in course content, teaching/learning modalities and assessments for mental health across Nepalese medical universities. The relative proportion of mental health in medical curricula as well as teaching/learning and assessments are far below ideal in these universities. These findings suggest a need for increasing time allocation, adopting newer teaching learning methods, and also having a mandatory clinical rotation during training and during internship.

## Background

Mental health is an important dimension of health as defined by the World Health Organization. Globally mental and substance use disorders account for 30 % of the non-fatal disease burden and 10 % of the overall disease burden [1] and this is projected to rise [2] in the years to come. Yet, the concern on mental health is not being adequately addressed resulting in a daunting treatment gap of 90 % in many parts of the world [3, 4]. In Nepal, more than 1 in 4 Nepalese are found to have either anxiety or depression or mixed anxiety and depression at any point in time [5]. Similarly, about one fourth of the people attending primary health centers have mental health problem [6]. Mental health disorders are responsible for 18 % of total disease burden due to NCDs which is equivalent to 7 % of total disease burden in Nepal [7]. But, only 1 in 10 of those having mental health problem get the service they need [8]. Similar to other low and middle income countries [9], there is just about one psychiatrist per 200,000 population in Nepal [10]. In order to fix this acute human resource shortage hindering service access, there is global movement to mobilize general health workers working at primary care level to provide basic mental health service. The unfolding global evidence strongly supports that mental health interventions delivered at the primary care level by non-specialist health workers are cost effective and feasible even at low resource settings [11]. Based on this evidence, the World Health Organization’s Mental Health Action Plan 2013–2020 [12] has called for integrating mental health services into primary care. Similarly, Nepal’s National Mental Health Policy [10] and multisectoral strategy for the prevention and control of Non-communicable Diseases (NCDs) [13] have also strategized to expand mental health services at the Primary Health Care (PHC) level.

The PHC service in Nepal is delivered predominantly by paramedical health workers and general medical doctors with a Bachelor in Medicine and Bachelor in Surgery (MBBS) degree (referred to here as MBBS doctors). The MBBS doctors are increasingly occupying primary health care centers (PHCCs) in Nepal since the government introduced a regulation requiring those educated under a government scholarship scheme to undergo 2 years compulsory rural service after graduation. Also, all the MBBS doctors must have at least one-year work experience to be eligible for the postgraduate training. These doctors are expected to provide a range of basic health services including mental health services from PHCCs. The quality of the (mental)health services provided by the MBBS doctors depends on their competence in medical knowledge, patient care, professionalism, communication and interpersonal skills, practice-based learning and improvement, and systems-based practice as identified by the Accreditation Council for Graduate Medical Education (ACGME) [14]. There is dearth of evidence on the competence of MBBS doctors to deliver basic mental health services at PHCCs in Nepal. A qualitative study done among primary care providers in Nepal reports that MBBS doctors are ill prepared to interview, diagnose and treat the mental health conditions owing to their lack of training and knowledge on the matter [15].

Ministry of health and population, Nepal (MoHP) has developed a 6-day additional training course on mental health for MBBS doctors in order to meet the demands of primary care and to maintain the quality of the services provided. Such trainings require health workers to travel leading to disruption in service as many health facilities depend on a single MBBS doctor. While such training is valuable to enhance the skill of the practising MBBS doctor, a robust mental health training during MBBS education could produce medical graduates with knowledge and skills required to deliver basic mental health services. It could further reduce the number of training days, which would ultimately reduce costs and, more importantly, increase the number of days health workers stay at their duty stations. Additionally, integrating mental health training in the curriculum could help to foster positive attitudes towards mental health, [16] reduce prejudice [17] associated with mental disorders and motivate more students to pursue psychiatry as their future profession [18]. The World Psychiatric Association (WPA) and the World Federation of Medical Education (WFME) also recommend that psychiatry should occupy a major part in the medical curriculum [19]. In the same principles, Sri Lanka has strengthened the psychiatry curriculum which has resulted in improvement in attitudes of final-year medical students towards psychiatry [20]. However, the mental health training curriculum of the MBBS degrees of Nepalese universities has never been critically analysed. Hence, this study aimed to review the MBBS mental health curriculum for medical students of all the medical universities in Nepal with respect to content coverage, teaching methods and evaluation patterns. This information will not only help us to assess the current status of mental health education, but also form the basis to advocate for updating the curricula proportionate to the disease burden and the need at the primary care level.

## Methods

### Study context

In Nepal, the MBBS is a 5.5 to 6-year university medical degree offered to high school graduates selected competitively based on merit. The training period includes first 2 years of Basic Sciences followed by clinical medicine and a mandatory one-year internship at the end after clearing examinations. The students undergo clinical rotations where case assessment, case presentation and clinical rounds are held, and the students undergo demonstration and practice of clinical procedures. The teachings are conducted in addition to clinical rotation. The internships rotations conducted at the end are intended for the graduates to practice the clinical work under supervision.

Medical education and medical practice are regulated by Nepal Medical Council (NMC), which administers licensing examinations for the medical graduates to register them as a bona fide medical doctor qualified to practice medicine independently. Since1978, when the Institute of Medicine (IoM) of Tribhuvan University (TU) started training MBBS students for the first time in Nepal [21], the number of medical schools [22] offering MBBS has grown to 20, operating under four universities: eight medical schools are affiliated to TU;10 to Kathmandu University (KU);and one each to BP Koirala Institute of Health Sciences (BPKIHS) and Patan Academy of Health Sciences (PAHS). This rise is largely due to an increase in the number of the private medical schools affiliated to TU and KU. All the medical schools follow the curriculum of their affiliating universities. The annual MBBS intake in these medical schools varies depending on the periodic assessment of the medical schools by NMC. For example, there were more than 2100 MBBS slots open in 2014 [23] of which 10–20 % were reserved for the government’s scholarship scheme. In addition to these, a number of students travel abroad to study medical sciences.

### Study procedure

This is a descriptive exploratory study. The study includes curriculum of all four Nepalese universities offering MBBS courses. Information on existing mental health curriculum from each university was collected from the faculty of the respective universities on March 2017. An expert team was formed with one psychiatry faculty member from each of the universities along with public health experts from WHO Nepal. The team developed a curriculum review tool by reviewing the current mental health curricula and medical education literature [24–27]. The tool broadly focused on the total time spent on mental health, teaching and learning methods used, content coverage, assessment modality and relative weight on mental health and the NMC requirements. Teaching and learning methods were classified broadly as passive or active. Lectures and demonstrations were considered passive methods while focus group discussions (FGDs), problem based learning (PBL), case based learning (CBL), student’s seminars, and research projects were taken as active methods [28, 29]. The clinical rotations are considered as case-based learning, while rotations during internship is separately presented as it is done after the completion of the study period. Assessment methods are classified based on the discussions in Ronald M. Epstein’s review article [30]. The content coverage of the curriculum was presented as patient assessment, the priority MH conditions, clinical psychology, the child mental health. Other topics included in the curriculum are listed separately (as mentioned in Table 1). The priority MH conditions are identified based on the WHO’s mhGAP Interventions Guide v2 [31] and Nepal’s Community Mental Health Care Package [32]. WHO’s mhGAP [31] identifies depression, psychosis, suicide, child and adolescent mental and behavioural disorders, dementia and disorders due to substance use as the priority mental health disorders. In addition to this, Nepal’s community mental health care package [32] included conversion disorder, somatoform disorder, and anxiety disorder as priority disorders in local context. The child mental health is separately presented to highlight the specific needs of child and adolescents while additional categories were added by the expert team. The NMC regulations for the medical schools were extracted from the documents in the web [22]. The compiled information was verified by the faculties of the respective universities.

**Table 1:** Content coverage of the curriculum

S.N	University	Content coverage and relative weight				
		**History taking and mental status examination**	Priority mental neurological and substance use disorders*	Behavioural science**	**Child mental health**	Other topics ***
	TU	√√	√√√	×	√	√√
	KU	√	√√	×	√	√
	BPKIHS	√√	√√√	√√	√	√
	PAHS	√√	√√√	×	√	√

*includes depression, mania and bipolar disorder, Psychosis, use of alcohol and other psychoactive substances, suicide, stress related and anxiety disorders, conversion and somatoform disorder, dementia and delirium and psychiatric emergencies. The emotional and behavioral problems among children is separately presented

** constitutes basic psychology lectures during basic science training in the first two year of training

*** includes classifications in Psychiatry, organic psychiatry, sleep cycle and sleep-wake disorders, community psychiatry, rehabilitation, neuropsychiatric aspect of chronic illness, psychosomatic disorders, psychosexual disorder, eating Disorders, mental disorders and disability, grief and bereavement

√: relative weight (maximum √√√ ) ×: None

## Results

The duration of teaching on mental health ranges from 25 h at KU to 92 h at PAHS. The clinical rotation for mental health is mostly 2 weeks, except at BPKIHS where it can go up to 4 weeks including a 2-weekelective clinical rotation. The percentage of marks of summative assessment on mental health out of total marks of the entire training ranges from 0.21 % at KU to highest 2.4 % at PAHS. Based on the time spent on mental health and the proportion of assessment marks allocated to mental health, PAHS scores highest closely followed by BPKIHS, TU and finally KU. BPKIHS, though it spends as much time as PAHS on teaching, dedicates only about 1 % of total examination marks to mental health. While the rest of the TU affiliated colleges have a mandatory two-week internship, BPKIHS provides up to 4 weeks of internship rotation, though two weeks of this is elective (Table 2).

**Table 2:** Mental health curricula of medical schools*

S.N.	University	Duration of teaching (hours)	Clinical rotation	Summative assessment marks (% of total course marks)	Internship duration
1	TU	45	2 weeks	40 out of 3300 **(1.2 %)**	1–2 weeks
2	KU	25	1–2 weeks	9 out of 1400 **(0.21 %)**	1 week
3	BPKIHS	58	2 weeks compulsory and 2 weeks elective	124 out of 12,000 **(1.1 %)**	2–4 weeks (Elective)
4	PAHS	92	2 weeks	24 out of 1000 **(2.4 %)**	2 weeks

*Duration of teaching hours, clinical rotation and internship may slightly vary across the affiliated medical colleges

All of the universities teach psychiatry predominantly through didactic lectures except for PAHS, where the curriculum is PBL based. BPKIHS and PAHS also use student seminars as another major teaching learning method. All curriculum has case-based learning included in the curriculum. Overall, BPKIHS and PAHS curricula are based more on a student focused active learning process, whereas those of TU and KU, covering 18 of the 20 medical schools, are based on traditional didactic lectures. BPKIHS and PAHS also provide opportunities to take up research projects in Psychiatry as elective (Table 3).

**Table 3:** Methods of teaching mental health in different universities

S.N	University	Methodology of teaching (relative weight)				
		Lecture	Problem based learning	Case based learning	Students’ seminar	Research projects
1	TU	√√√	×	√	×	×
2	KU	√√√	×	√	×	×
3	BPKIHS	√√√	√	√	√√	√ (Elective)
4	PAHS	√	√√√	√	√√	√ (Elective)

√: relative weight (maximum √√√ ) ×: None

All the universities show a relative focus on priority mental and substance use disorders as defined in the WHO’s mhGAP [29] and the National Community Mental Health Care Package 2017 [30]. It is notable, however, that TU’s curriculum is thinly spread to cover a range of topics other than priority conditions, in contrast to other universities. On the other hand, only the curriculum of BPKIHS emphasizes behavioural science which constitutes basic psychology lectures during basic science training in the first two year of training. Attention to child mental health is minimal across all the universities (Table 1).

BPKIHS and PAHS carry out formative assessment in addition to summative assessment while KU and TU carry out summative assessment only. However, in all the universities, mental health is combined as into other papers such as Internal Medicine (in KU and TU) or a paper consisting of Dermatology, General Practice, Lab Medicine and Radiology. The proportion of mental health in the combined paper ranges from 3.75 % at KU to 31 % at BPKIHS.TU requires students to present cases during exams, while BPKIHS and PAHS have stronger component of simulated clinical assessment. KU on the other hand has no skills assessment in the examination for mental health. No university requires students to separately pass the psychiatry sections in combined paper except TU where students have to pass practical assessment separately (Table 4).

**Table 4:** Assessment pattern of mental health

S.N.	University	Assessment pattern			
		**Assessment modality**	**Assessment methods**	**Separate subject**	**Proportion of Psychiatry in the combined paper**
1	TU	Summative	Written examination - Multiple choice questions - Short answer questions Direct Observation - Clinical short case	Partially (theory as part of internal medicine and practical a separate paper)	40 out of 400 (10 %)
2	KU	Summative	Written examination - Multiple choice questions - Short answer questions	No (as a part of internal medicine paper)	9 out of 240 (3.75 %)
3	BPKIHS	Formative and Summative	Written examination - Multiple choice questions - Short answer questions - Modified essay questions Clinical Simulation - OSCE Viva (oral examination)	No (a combined paper consisting of Dermatology, General Practice, Lab Medicine and Radiology)	124 out of 400 (31 %)
4	PAHS	Formative and Summative	Written examination - Multiple choice questions - Short answer questions - Modified essay questions Clinical Simulation - Standardized patient - OSCE Directly Observation - Mini case based discussion (Mini CEX Viva (oral examination)	No Combined with other subjects (Integrated) called as subjects for short rotation	24 out of 200 (12 %)

Nepal Medical Council (NMC), the governing body of medical education in Nepal, requires medical schools to have a separate Psychiatry department with daily out-patient and in-patient service (Table 5). There are no clear requirements for the content to be covered, duration of theoretical teaching, clinical rotation, assessment modality or internship requirements. The NMC licensing examination allocates only 1.1 % of total assessment marks to mental health (Table 5).

**Table 5:** Mental health requirements of the Nepal Medical Council [33]

Areas	Requirement
General requirement	For 75–100 students: a psychiatry department with 3 faculty members and an inpatient unit with 30 beds For 50–75 students: a psychiatry department with 2 faculty and an inpatient unit with 10 beds
Content to be covered	Not specified
Duration of theoretical teaching	Not specified
Duration of clinical rotation	Not specified
Examination pattern	Not specified
Internship requirement	Not specified
Coverage of mental health NMC licensing Examination	2/180 = 1.1 %

## Discussion

This study presented the mental health curricula of MBBS of all medical universities in Nepal with respect to content coverage, credit hours, clinical rotation, examination methods and marks, internship rotation. All four medical universities in Nepal currently include mental health in their MBBS training curricula but there are considerable discrepancies in content coverage, credit hours, clinical rotations and assessments. Traditional didactic lectures are the main methods of teaching in two universities with a most of the affiliated medical schools. The relatively new medical schools (BPKIHS and PAHS) have adopted active teaching and learning methods such as student seminars and the PBL. Nepal Medical Council, the regulating body of medical education and practice, though it requires each medical school to have a psychiatry department; however, does not have explicit requirements on the duration of teaching, teaching content, assessment methods or the clinical or the internship rotation relating to mental health. And NMC’s licensing examination contains negligible content on mental health.

The amount of mental health teaching is not commensurate with the disease burden these disorders pose. Mental health occupies less time for teaching (25–92 h) and the clinical rotation (3–5 weeks including internship).This under prioritization on mental health training is a global issue [32, 33]. The curriculum in Nepal is comparable to the requirement by the national medical council in India and Bangladesh. The Bangladesh Medical and Dental Council (BMDC) mandates 20 h didactic lectures and 3-week clinical rotation ward placement in the 3rd phase for clinical classes in psychiatry with no mandatory placement in psychiatry during the internship preparation of the last one year [34]. The Medical Council of India has recently increased the teaching hours from 20 to 40 h; the clinical posting from 2 to 4 weeks, and mandated the compulsory answering of the exam questions, internal assessment and obligatory Psychiatry posting in the internship [35]. In Sri-Lanka, however, Psychiatry teaching program involves a total of 8–12 weeks of clinical training and 40–75 h of lectures with additional tutorials and seminars and has been recognized as one of the five major subjects in the final examination, on par with medicine or surgery [20].

Similarly, Psychiatry has received better attention in developed countries such as the United States and United Kingdom. In US the Harvard Medical School for example has a preclinical course Mind, Brain, and Behavior and the Core Psychiatry Clerkship for 4 weeks in addition to offering a wide range of psychiatry electives [36]. In the UK, the General Medical Council (GMC) has emphasized the psychological and behavioral principles in patient care in a whole and to provide appropriate support to people with mental illness [37].

The limited time allocated to mental health has been spent on less efficient teaching learning methods at large universities. There is opportunity to adequately focus the curriculum on the priority mental health and substance use disorders as identified in the mhGAP modules [31] and in the Nepal’s Community Mental Health Care Package [32]. Child mental health is largely ignored in all the curricula despite the fact that 42 % of the total population is child and adolescents [38] and 10–20 % of them will have some mental health issues [39]. A considerable time is spent on thinly covering all the disorders without a special focus in one of the universities. Similarly, rather than adopting student centered active teaching learning methods, didactic lecture is the main method of teaching psychiatry in the larger universities with affiliated medical schools. Lectures are useful to cover a substantial amount of information and concepts within a relatively short period of time in the academic calendar [29]. However, they do not effectively enable a student-centered learning or bring about the expected changes in the behaviour of students because students are regarded as passive listeners [29]. Non the less there have been attempts (in younger medical schools) to adopt relatively newer teaching and learning methods which are student centered and active making students assume a sense of responsibility for the content provided to them. Lectures of BPKIHS are framed as Structured Interactive Sessions (SIS), requiring teachers to incorporate more interactive components in an attempt to engage the students in the lessons, while PAHS adopting PBLs and student’s seminar.

Assessment plays an important role in motivating and directing medical students to future learning and also in protecting the public by identifying incompetent physicians. Ideally, assessment of competence should provide insight into actual performance in the clinical setting as well as capacity to change and generate new knowledge [40]. As all forms of assessment methods have strengths and limitations [30], it is the optimal mixture of different assessment methods that could meet the objectives of the assessment. This ideology has been reflected in training curriculum of one of the medical school (PAHS) which has introduced Mini Clinical Evaluation Exercise (Mini-CEX) [41] and Directly Observed Practical Skill (DOPS) [42], as well as other peer assessment to their routine evaluations, in a bid to reflect the complexity of real life medical scenarios. This medical school also conducts formative assessment to orient the students as they approach a relatively unstructured body of medical knowledge [30]. Formative assessments can reinforce students’ intrinsic motivation to learn and inspire them to set higher standards for themselves [43]. On the contrary, examination on mental health in KU affiliated medical colleges is based on written question answers which only assess knowledge. TU affiliated colleges, in addition to knowledge based written examinations, require students to present a short case to the examiners. BPKIHS has a combination of theory and practical assessments with inclusion of both formative and summative assessments. The theory assessment includes a combination of multiple-choice questions, short answer questions, structured long answer questions and modified essay questions as well as assessments of seminars to assess both the cognitive as well as affective domains of learning. The practical assessments include a viva and observed structured clinical examinations(OSCE) [44] to assess different domains (cognitive, psychomotor and affective) of learning. However, there is no provision of short or long case presentations at BPKIHS. Across the medical colleges, the proportion of mental health in overall assessment is relatively low, such that students can pass the overall summative assessment requirement even without attempting mental health examinations. When assessment modalities do not require that students pass the subject, this can weaken students’ motivation that particular subject, as neither the ‘push’ nor the ‘pull’ force seems to be operational [45]; it seems to be true particularly in the KU affiliated colleges. This may further influence the attitude of medical students towards psychiatry, such that students are less likely to pursue a future career as psychiatrist [18] .

The WPA and the WFME, through a core curriculum committee, have developed detailed guidelines for the “Core Curriculum in Psychiatry for Medical Students” [19]. None of the medical curricula in Nepal meets the recommendations set forth in these guidelines. None the less, each of the curricula has certain strengths for the rest to learn from. The student-centered active learning methods the PAHS is following can be an important lesson for other universities in order to efficiently use the limited time given to mental health. Similarly, the comprehensive assessment modalities of PAHS can be adopted by other universities, while the requirement to separately pass the practical examination in TU can be important lessons for the rest of other medical universities. The special focus on behavioural science at preclinical years at BPKIHS curriculum can be something for the rest of the universities to follow. Moreover the medical universities are yet to adopt the competency based curriculum in the undergraduate medical education despite the growing global consensus [46, 47]. The competency based framework emphasizes more on the clear indented outcome and learner centeredness than on the learning strategies or the formats or the time-based training [46, 47]. More importantly, Nepal Medical Council, the regulating body of medical education and practice, does not have explicit requirements on the outcome or the strategies of the teaching and learning in mental health in line with this global development.

## Conclusions

The importance of mental health and psychiatry as a distinct subject in the curriculum of MBBS has been realized by all the universities of Nepal. However, there exist considerable disparities in course content, teaching/learning modalities and assessments across all universities. In addition, the proportion of mental health in the broader curriculum as well as teaching/learning and assessments are far below ideal in all the four universities. To strengthen community mental health facilities in general, and to develop positive attitudes towards mental health and mental illnesses among medical students in particular, the current curriculum is not sufficient.

## Recommendations

Based on the findings of this study, we recommend that the concerned universities and the NMC revise existing curricula in line with the recommendations of the guidelines for the “Core Curriculum in Psychiatry for Medical Students” [18] (with adaptation for Nepal’s local context)to increase the priority given to mental health. This will require increasing time allocation for mental health teaching, adopting newer teaching learning methods such as student-centered approaches with active learning components, and implementing a mandatory clinical rotation during training and during internship. As the newer thoughts on medical education is that it should be competency based, so we would emphasize this model to be incorporated in the curriculum of all the universities. These changes will enable MBBS doctors to meet the huge patient load they will encounter when posted at primary care health facilities across Nepal. Mental health should be assessed separately in medical examinations with the requirement that this to be passed as a separate subject during the summative assessment. NMC has a critical role to play in developing regulations in consultation with the medical schools in order to implement the above recommendations.

## Strengths and limitations

This is first ever study in Nepal to look into medical curriculum and make comparison to sort out “mental health” component. We have provided a broad overview of mental health curricula of four medical universities of Nepal. Although, medical schools are expected to follow the curriculum of the universities they are affiliated with, there are always some discrepancies in practice. We might not have captured different approaches the affiliated medical schools were following in this paper. We also have been unable to capture the amount of teaching on behavioural science and psychosocial aspects of medicine integrated into other disciplines such as General Practice or Internal medicine. Additionally, this paper does not incorporate the quantitative information on the competency of the medical graduates on mental health on providing services to people with priority mental health problems.

## Data Availability

The information analysed in the study is obtained from the faculties of respective universities, who are also the co-authors of the paper. These can be found in the main curriculum of the MBBS programme of the respective universities.

## Abbreviations

ACGME: Accreditation Council for Graduate Medical Education
BPKIHS: BP Koirala Institute of Health Sciences
CBL: Case Based Learning
DOPS: Directly Observed Practical Skill
FGD: Focus Group Discussion
KU: Kathmandu University
MCQ: Multiple Choice Questions
mhGAP: Mental Health Global Action Programme
Mini-Cx: Mini Case Based Discussion
MoHP: Ministry of Health and Population
NCD: Noncommunicable Disease
NMC: Nepal Medical Council
OSCE: Objective Structured Clinical Examination
PAHS: Patan Academy of Health Sciences
PHCC: Primary Health Care Center
PBL: Problem Based Learning
SIS: Structured Interactive Session
TU: Tribhuvan University
WFME: World Federation of Medical Education
WHO: World Health Organization
WPA: World Psychiatric Association
